# Identifying research priorities for health professions education research in sub-Saharan Africa using a modified Delphi method

**DOI:** 10.1186/s12909-020-02367-z

**Published:** 2020-11-18

**Authors:** Susan C. Van Schalkwyk, Elsie Kiguli-Malwadde, Jehan Z. Budak, Michael J. A. Reid, Marietjie R. de Villiers

**Affiliations:** 1 Centre for Health Professions Education, Faculty of Medicine and Health Sciences, Stellenbosch University, Stellenbosch, South Africa; 2 Health Workforce, African Centre for Global Health and Social Transformation (ACHEST), Kampala, Uganda; 3 Division of Infectious Diseases, Department of Medicine, University of Washington, Seattle, Washington USA; 4 Division of HIV, Infectious Diseases, and Global Medicine, University of California San Francisco, San Francisco, USA; 5 Division of Family Medicine and Primary Care, Faculty of Medicine and Health Sciences, Stellenbosch University, Stellenbosch, South Africa

**Keywords:** Health professions education, Research, Delphi method, Africa

## Abstract

**Background:**

Recent increases in health professions education (HPE) research in sub-Saharan Africa (SSA), though substantial, have predominantly originated from single institutions and remained uncoordinated. A shared research agenda can guide the implementation of HPE practices to ultimately influence the recruitment and retention of the health workforce. Thus, the authors aimed to generate and prioritise a list of research topics for HPE research (HPER) in SSA.

**Methods:**

A modified Delphi process was designed to prioritise a shared agenda. Members of the African Forum for Research and Education in Health (AFREhealth) technical working group (TWG) were asked to first list potential research topics. Then, members of the same TWG and attendees at the annual AFREhealth academic symposium held in Lagos, Nigeria in August 2019 rated the importance of including each topic on a 3-point Likert scale, through two rounds of consensus seeking. Consensus for inclusion was predefined as ≥70% of respondents rating the topic as “must be included.”

**Results:**

Health professions educators representing a variety of professions and 13 countries responded to the survey rounds. Twenty-three TWG members suggested 26 initial HPER topics; subsequently 90 respondents completed round one, and 51 completed round 2 of the modified Delphi. The final list of 12 research topics which met predetermined consensus criteria were grouped into three categories: (1) creating an enabling environment with sufficient resources and relevant training; (2) enhancing student learning; and (3) identifying and evaluating strategies to improve pedagogical practice.

**Conclusions:**

Establishing research priorities for HPE is important to ensure efficient and appropriate allocation of resources. This study serves as a reminder of how the prevailing context within which HPE, and by implication research in the field, is undertaken will inevitably influence choices about research foci. It further points to a potential advocacy role for research that generates regionally relevant evidence.

**Supplementary Information:**

The online version contains supplementary material available at 10.1186/s12909-020-02367-z.

## Introduction

There has been a rapid increase in the number of health professions training institutions in Sub-Saharan Africa (SSA) in order to train more health professionals for the region. Despite this, SSA remains challenged in meeting the health needs of its populations, exacerbated by existing and emerging epidemiologic challenges [1]. There has, however, been global interest in strengthening human resources for health (HRH) in the region. Multiple initiatives to improve both the number of graduate outputs and the quality and relevance of their training have been launched [2]. One example is the Medical Education Partnership Initiative (MEPI) and Nursing Education Partnership Initiative (NEPI), a $130 million competitively awarded grant by the President’s Emergency Plan for AIDS Relief (PEPFAR) to medical and nursing schools in 12 Sub-Saharan African countries from September, 2010 to August, 2015. The goals of MEPI and NEPI were to increase the capacity of the awardees to produce more and better doctors and nurses, strengthen locally relevant research, promote retention of graduates within their countries, and ensure sustainability [3]. The establishment of the African Forum for Research and Education in Health (AFREhealth), in 2017, has now sustainably consolidated these initiatives. AFREhealth aims to collaborate with stakeholders to improve health outcomes, work towards an AIDS-free generation, establish a research agenda for health priorities in Africa, and mobilise vital resources [4].

Much of the published material regarding research in Africa has cited the need to establish a coordinated research agenda to inform priorities [5]. This is equally true for health professions education research (HPER), which is critical to the success of health professions education (HPE) centers and departments [6, 7]. Relatively little has been published on HPER in SSA, despite the increase in the number of training institutions. Although there has been an increase in research outputs in recent years, assisted by the establishment of the African Journal for Health Professions Education (AJHPE), this increase has not matched the rapid growth in the number of institutions. During MEPI, over 376 peer-reviewed publications, including a special supplement in Academic Medicine (2014), were published, however most of these articles tended to be descriptive in nature [3]. Van Schalkwyk [8] highlighted the need for strengthening research capacity to generate a wider evidence base in HPE, moving beyond description to contributing to theory building in the field [9]. As a first step towards responding to this call, we sought to establish what role players in the region would regard as priorities for HPER initiatives. An assumption was that having a shared agenda can strengthen collaborative work in the field and can contribute to appropriate allocation of resources. In addition, and given the importance of context in improving health and the delivery of HPE, local research can also identify potential challenges, set priorities, devise original solutions, and make the best use of scarce resources [10, 11]. Therefore, through leveraging the input of regional experts and researchers, we sought to identify a set of HPER priorities to facilitate the implementation of regionally relevant initiatives and concomitantly guide resource allocation.

## Methods

We used the Delphi method (with modifications) to establish priorities for HPER in SSA. The Delphi method is a consensus-building approach which seeks expert opinion on a pre-determined topic in a structured and iterative manner [12, 13]. The Delphi method can be useful in areas where evidence-based literature is limited, as it can unearth collective knowledge from those in the field [13–15]. A series of rounds are used to clarify, refine, and ultimately achieve consensus on the area under discussion. A key feature of the method is that participants or respondents provide input independently and anonymously during each round, resulting in a process that is not unduly influenced by any one individual or subset of respondents [12, 16].

The Delphi method usually involves six steps, namely the identification of a research question/problem; conducting a literature search; developing a set of statements around the topic of choice; performing anonymous iterative rounds; providing feedback to the respondents between rounds; and summarising the findings [14]. We report our process accordingly.

### Step 1 – identifying the research problem/question

**Fig. 1:**
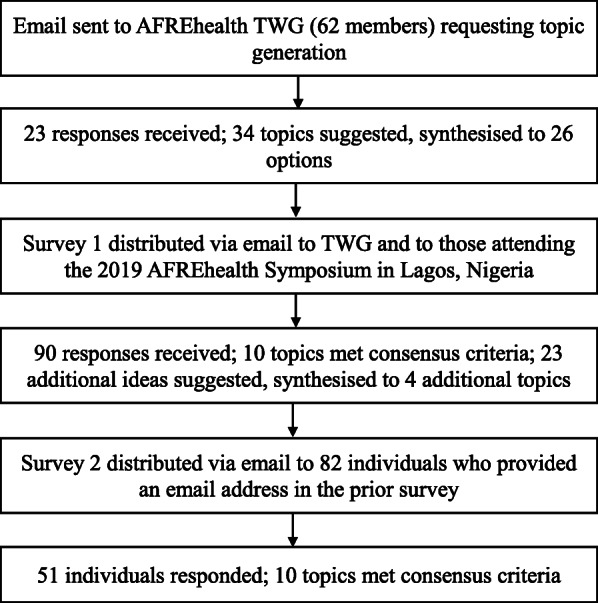
Flow diagram documenting the Delphi process

Our process involved one round of item generation and two rounds of consensus seeking carried out between April 2019 and October 2019. A group of researchers involved in the AFREhealth network led the process. The authors formed a study group that refined the research question over several project meetings and discussions. Three of the authors (SvS, EKM, MdV) are practicing health professions education experts based in Africa. The other two team members (MR and JB) are infectious diseases experts with a special interest in health professions education, based in the USA. The consensus development process is summarised in Fig. 1.

### Step 2 – literature search

To inform the development of the protocol for our study, we explored the relevant literature, including work describing the Delphi methodology, and studies that have sought to establish research priorities for their specific contexts [11, 14–16].

### Step 3 – topic generation

A core group of 62 AFREhealth educators and investigators, members of the AFREhealth HPER Technical Working Group (TWG) comprising colleagues from the region with an expressed interest in HPER, were invited to generate topics for consideration. In April 2019, we sent via email a survey designed in Qualtrics© (Provo, UT) [17] asking, ‘*what are possible research questions, topics, or areas of focus that should be a priority in a health professions education research agenda for sub-Saharan Africa?’* We also requested descriptions and rationales for each research question proposed. Two email reminders were sent. Twenty-three people responded proposing an initial list of 34 research priorities. This list was synthesised by two of the researchers (SvS and MdV) to remove unnecessary duplication, resulting in a list of 26 items. At this point, the research team made a decision to rephrase all of the items as priority topics to ensure consistency across the items.

### Step 4 –conducting two iterative anonymous rounds

The list of 26 research priorities generated during stage 3 were distributed in the form of a survey for prioritisation to members of the working group, as well as to a broader group of researchers and stakeholders who attended the AFREhealth Symposium in Lagos, Nigeria in August 2019. This step (Round One) represents a modification in our Delphi in that we introduced additional respondents after the topic generation stage. This meeting involved researchers and educators in the health professions, as well as service providers in the field, mainly from across Anglophone sub-Saharan Africa. Those attending the symposium were asked to complete the prioritisation survey either online (designed in Qualtrics©) or using a paper-based version. Those members of the TWG not present in Lagos were invited via e-mail to complete the same online survey. Two email reminders were sent. Ninety individuals participated in this first round to begin prioritising the topics generated in step 3. Basic demographic information was requested from respondents. Responses were anonymous and treated confidentially. All respondents were asked to rate how important each of the 26 research topics would be to include in a HPER research agenda for SSA using a 3-point Likert scale that ranged from ‘do not include’ to ‘could be included’ to ‘must be included.’ Respondents could also pick ‘no vote,’ if they felt they did not have enough knowledge on a topic to make an informed decision of its importance. Those votes were coded as null. Topics were itemised in random order. Consensus criteria, that only topics rated as ‘must be included’ by ≥70% of respondents would be included, were set a priori. In addition, an open-ended question prompted submission of additional research topics or agenda items in this round. New topics were reviewed and consolidated by the research team into existing themes or added to the list.

For Round Two, in September 2019, all respondents who provided their e-mail addresses in Round One were sent a link via email to the final online survey in Qualtrics©. The final survey included 10 topics which had met consensus criteria in Round One, and 4 additional suggested topics, generating a total of 14 topics. In addition, they were given feedback on the first round. Respondents were again invited to rate each research priority on a 3-point Likert scale in terms of importance to include with consensus set at ≥70% of respondents who rated the topics as ‘must be included. Topics were itemised in random order. After Round Two, sufficient consensus was reached on a smaller number of topics [10] for the process to be terminated.

### Step 5 – providing feedback to respondents

Round One respondents who provided their email addresses received feedback. This feedback was included in the email request to participate in Round Two and outlined the areas that achieved consensus in the first round, in addition to including the percentage of respondents rating each top as ‘must be included,’ as well as the additional items that were generated in Round One.

### Step 6 – summarise the findings

The research team, first individually and then collectively, thematically grouped the items that achieved consensus in three areas, thus applying a next order round of analysis. These are reported in the Results section below.

In sum, Table 1 reports the quality criteria for Delphi studies as proposed by Humphrey-Murto et al. [14], with three additional criteria from Diamond et al., [12] as applied in our study.

**Table 1:** Quality characteristics of this study [12, 14]

Quality characteristic	Our Study
Literature review conducted	Yes
Background information provided to respondents	Yes
Purpose is item generation or ranking or both	Yes
Number of respondents indicated	Yes
Number of respondents for round 1 indicated	Yes
Number of respondents for round 2 indicated	Yes
Were criteria used for respondents reproducible^a^	No
Polling described	Yes
Private decisions collected (anonymity)	Yes
Formal feedback of group ratings	Yes, after round 1
Number of rounds conducted 2 or more	Yes
Number of rounds determined a priori	Yes
Predetermined definition of consensus	Yes
Consensus forced	Yes
Were criteria used for dropping items clear?^a^	Yes
Stopping criteria other than rounds specified?^a^	Yes

^a^indicates criteria from Diamond et al

The protocol for this project was reviewed and approved by the University of California, San Francisco’s Institutional Review Board (IRB) in San Francisco, California. Consent was considered implied by participation in the study as approved by the IRB (#19–28,050).

## Results

Twenty-three individuals from the HPER TWG participated in topic generation. Ninety responses were received in Round One. For Round Two, the survey was sent via email to the 82 individuals from Round One who provided their email addresses. We received 52 responses (52/90, 58% response rate) in Round Two. Round One’s respondents were from thirteen countries in SSA, with the majority from Nigeria, South Africa, and Uganda. In Round Two, 23/52 (44%) of the respondents were from Nigeria, 10/52 (19%) were from South Africa, and 7/52 (13%) were from Uganda. (Table 2) To account for the fact that the majority of respondents in both rounds were Nigerian, an additional sensitivity analysis was performed to determine if research priorities were different among respondents from Nigeria compared to elsewhere in SSA. The final list of priority research issues was the same among Nigerian respondents as compared to respondents from other countries.

**Table 2:** Country of primary practice of survey respondents by round

Country	Round 1 Respondents (***n*** = 90)	Round 2 Respondents (***n*** = 52)
Democratic Republic of Congo	5	1
Ethiopia	7	6
Ghana	1	0
Kenya	2	0
Lesotho	1	1
Namibia	2	1
Nigeria	38	23
South Africa	11	10
Tanzania	2	1
Uganda	8	7
Zambia	2	2
Zimbabwe	2	1
United States	1	0

Respondents reported a median of 14 years’ experience working in HPE, with a range from one to 42 years. Although the majority of the respondents were medically and nursing qualified, the individuals represented a wider range of health professions as well as a few others (Table 3).

**Table 3:** Occupations of survey respondents by round

	Round 1 Respondents (***n*** = 90)	Round 2 Respondents (***n*** = 52)
Health professional		
Dentist	1	1
Medical doctor	36	23
Nurse	14	11
Occupational therapist	1	1
Pharmacist	6	4
Physiotherapist	1	1
Psychologist	1	0
Public health	2	2
Scientist	3	2
Other	2	2
Not a health professional		
Accountant	1	1
Anthropologist	1	0
Education specialist	2	2
Health economist	1	0
Health manager	1	0
Information technology	1	0
Law	1	0
Research administrator	1	1
Sociologist	1	0

In Round One, 71/90 (79%) of respondents regarded themselves as experienced or somewhat experienced in HPE, with 25/90 (28%) having published more than five articles in the field. In Round Two, 41/52 (79%) of respondents regarded themselves as experienced or somewhat experienced in HPE, with 25/52 (48%) having published more than five articles in the field (Table 4).

**Table 4:** Characteristics of survey respondents by round

	Round 1 Respondents (***n*** = 90)	Round 2 Respondents (***n*** = 52)
Health professional		
Yes	71	47
No	11	5
Experience in HPER		
Novice	19	11
Somewhat experienced	46	23
Experienced	25	18
Number of HPE-related publications		
None	35	18
Less than 5	30	9
More than 5	25	25
No answer	2	–

*HPER* Health professions education research, *HPE* Health professions education

Thirty-four topics and topic descriptions were generated by the TWG members, which were reduced to 26 topics by the research team (see previously). In Round One, ten topics met consensus criteria. Twenty-three new topics were suggested, and four of these were incorporated into the next round; those that were not included were deemed sufficiently similar to ones already included in the existing list. In the second topic prioritisation round, ten topics met consensus criteria. (Table 5).

**Table 5:** Final list of topics in order from highest consensus rating to lowest

Rank order	Topic	(%) Rating “Must Include”
1	Addressing the human resources for health challenges in rural and remote areas	98
2	Interprofessional collaboration and practices in SSA	95
3	Teaching a holistic and person-centered care approach	92
4	The role of information communications technology in HPE	85
5	Faculty development for clinical teaching	82
6	Quality assurance processes and procedures in health professions education	80
7	Resources, political commitment, and funding for HPE in SSA	76
8	Responsive curricula to the health needs of SSA	75
9	Potential of rural communities as platforms for training health care professionals	71
10	Relevance of communication skills training in culturally diverse contexts	70

### Synthesis of topics

The process of synthesis that occurred as a result of establishing consensus within the Delphi saw interesting shifts in perspectives from the initial set of 26 topics, to the final list of ten priorities. The early list, for example, included more generic issues such as ‘assessment practices’, ‘student retention’, ‘graduate competencies’, ‘post-graduate training’ and ‘self-regulation skills’ – topics that would likely resonate with health professions educators all over the world. The final list, however, represents what could be regarded as higher-order topics which, with one or two exceptions, speak to national and regional issues. They reflect the context within which health professionals in SSA are being trained and the challenges that characterise this context. During the topic generating phase of the study, the rationales that respondents provided in support of their selections emphasised the need for understanding ‘our own’ challenges in order to ‘decolonise our way of teaching and assessment.’ It was also argued that existing frameworks had been generated in ‘developed countries’ acknowledging the need for locally, relevant and responsive research. Ultimately, second-tier analysis by the research team resulted in three over-arching, but inter-connected themes: (1) creating an enabling environment with sufficient resources and relevant training; (2) enhancing student learning; and (3) identifying and evaluating strategies to improve pedagogical practice. These could be regarded as a triumvirate of educational endeavors – the teaching (pedagogy), the learning, and then the environment (context) within which it should occur. The themes are discussed below, supported in some instances with direct quotations from some of the rationales provided during the topic generation phase, and with several priorities having relevance across more than one.

#### An enabling environment

A key message from this Delphi is that a first step to defining and addressing the HPER priorities in SSA is to create the environment that will foster researchers, with sufficient training and resources to address the most pressing questions. This thesis is underscored by the fact that the top 10 priority topics include three that are focused on understanding the factors that currently make HPER challenging in SSA:

#### Priority # 1 – addressing the human resources for health challenges in rural and remote settings

This first ranked topic focuses on addressing human resources for health challenges in rural and remote settings. This may be an unexpected result for colleagues around the world, but needs to be seen in the context of HPE in SSA as described in the introduction and points to the lived experience of many educators in the field who practice their teaching in resource-constrained contexts. The importance of the environment in influencing teaching and learning has been well-documented [18]. It can be inferred that for our respondents, addressing the need for practitioners who can respond to the increasing burden of disease, particularly in rural areas, is a non-negotiable imperative whether in terms of the taught curriculum (as many medical schools and health sciences faculties embrace distributed clinical learning [19]), or in terms of ensuring an environment in which teaching can meaningfully occur. Linked to this was the underlying premise of providing quality health care for all.

#### Priority # 5 – faculty development for clinical teaching

Supporting those responsible for teaching, specifically clinical teaching, ranked fifth. Recommendations spoke specifically to looking to discern the status of faculty development in the region, describing it as a particular area requiring further investigation. Given the current drive towards the professionalisation of the educational role, and the growing need for faculty who can teach the growing numbers of HPE students, this focus was expected as there has been limited work in this area. An exception is research into the role of emerging clinical teachers which has been conducted in the region in recent years, [20, 21] with studies emphasising the need for further work and ongoing support for those responsible for HPE students in clinical training, particularly those who are placed in rural or distributed sites.

#### Priority # 7 – resources, political commitment and funding for HPE in SSA

This priority picks up on the higher order focus established in Priority # 1, foregrounding the economic and political instability that characterises much of the SSA region, the impact that this has on health care and the subsequent effect on HPE. During topic generation, respondents felt that education, research and service delivery activities should all be directed, “towards addressing priority health concerns of the community, region and/or nation that they have a mandate to serve.”

#### Enhanced student learning

Among the priorities identified, four foregrounded approaches could potentially enhance student learning. An important proviso for these priorities was that the ultimate aim of enhanced student learning was the delivery of graduate professionals who could respond to local and regional health care imperatives and provide quality health care.

#### Priority # 2 – Interprofessional collaboration in clinical practice in SSA

Priority #2 highlights an essential approach to healthcare training and practice, namely interprofessional education and collaborative practice (IPECP), that should inform the student learning experience. IPECP is currently foregrounded in HPE research, visible in a plethora of publications [22]. In the rationales provided for interprofessional collaboration during the topic generating phase of this study, respondents spoke directly to the under resourced context within which many in SSA work, and the extent to which collaboration across all healthcare practitioners will be crucial to address the workforce challenges identified under Priority # 1 *including* that it would enhance patient care. It should be noted that the establishment of a SSA organisation dedicated to fostering IPECP (The African Interprofessional Education Network (AfrIPEN): https://afripen.org/) in 2017 may account for why this particular topic was ranked so high. AfrIPEN has an affiliate relationship with AFREhealth, and it is plausible that some of our respondents are members of both organisations. In their rationales, however, respondents also made reference to the ‘African patriarchal social system’ that needs to be problematised as it is ‘contrary to the philosophical underpinnings of shared leadership, shared decision-making etc.’ that are so needed in the region.

#### Priority #9 – potential of rural communities as platforms for training health care professionals

Linked strongly to the need to address human resources for health challenges was a focus on enhancing student learning through distributed clinical training, specifically in rural areas. This priority emphasised the growing awareness of how large academic hospitals are not necessarily the best environment for training students to meet community needs.

#### Priority # 3 – teaching a holistic and person-centered approach and priority # 10 – relevance of communication skills training in culturally diverse contexts

Teaching person-centered and holistic care underscores the value of a comprehensive approach to the person in the African context which also takes into account the person’s values and needs, as well as their family and community [23]. A further indicator of the complexity of context can be seen in priority # 10 which recognises the need for students to be trained to communicate effectively with patients across multiple contexts and different cultures. This links with previously mentioned perspectives of respondents who felt it was important to support research initiatives that would provide responses for local and regional contexts.

#### Identifying and evaluating strategies to improve pedagogical practice

A final group of priorities, which includes some already mentioned above, relates to identifying and evaluating strategies to improve pedagogical effectiveness or assess new modes of curricula and pedagogical innovation.

#### Priority # 4 – the role of information communications technology in HPE

It could be argued that a focus on the role of information communications technology (ICT) in HPE would have been expected given the ubiquitous nature of blended and e-learning approaches in modern-day HPE [24]. Interestingly, however, this topic did not feature in the topic generating process, but was introduced in Round One and validated in Round Two. There can be no doubt as to the importance of this research focus, particularly investigation that can explore options for drawing out the affordances of ICT amid resource constraints. For example, while many rural areas in SSA may be isolated in terms of connectivity, the region is known for its high rate of cellular telephone coverage providing a lifeline for health care workers in these remote regions. Investigating the potential for HPE using mobile technology could, therefore, have particular relevance [25].

#### Priority # 6 – quality assurance processes and procedures in health professions education

This priority demonstrates the intent of respondents to ensure that educational practices and teaching innovations are carefully monitored and evaluated. Responses provided during the topic generation phase suggested that this focus has to do with being accountable given expectations by stakeholders for the provision of quality education.

#### Priority # 8 – determining how to develop and implement curricula that are responsive to the health needs of SSA

In 2010, the Lancet commissioned article exploring *Health professional in the twenty-first Century*, [26] argued that curricula had not kept pace with community healthcare needs, catalysing introspection among health professions educators and curriculum developers across the world. Given SSA specific resource constraints, and its unique burden of disease, exploring what a responsive curriculum for the region might look like would appear to be of significant value for the HPE community. It could be argued that this priority possibly provides a research focus that could encapsulate most if not all of the others that made the final list.

## Discussion

Establishing research priorities for HPE at a national or higher level is important to ensure maximum impact of efforts. Related work has previously been conducted in countries such as Canada [27], New Zealand [28], and Scotland [11], catalysed by a desire to foster collaborative and coordinated approaches to research [28] and to ensure targeted allocation of increasingly scarce resources [11]. Comparison between our results and those obtained in these studies highlights both similarities and differences. For example, issues of student access and selection, the role of assessment and feedback, resilience and well-being, phases of transition across curricula, amongst others, are strongly foregrounded in these earlier studies, but do not make it to our final list even though some featured in earlier iterations. The importance of faculty development is, on the other hand, one area of congruence, as is the importance of interprofessionalism. The key difference, however, resides in the extent to which the described need for political and economic stability, and the imperative to ensure suitably trained health professionals who can respond to national and regional challenges, is relatively silent in this earlier work, although there is reference to change management and the importance of leadership to facilitate such change.

What does this study mean for HPER in SSA going forward? What our Delphi highlights is that it is precisely the factors that currently undermine HPER activities in the region that have emerged as research priorities. Thus we are faced with a conundrum on a number of levels. Firstly, the identified set of priorities does not reflect specific research gaps, but rather points to respondents’ perception of challenges within HPE in general, emphasising the complexity of the context in which the training of future health professionals much occur. We acknowledge that this is probably linked to the fact that many of the respondents were clinicians and researchers with an interest in HPER, but not necessarily experts in the field itself. It could be argued that research activities per se are unlikely to impact the national political and economic structures that currently determine the different health systems represented in the study, but perhaps that advocacy work, strengthened by locally generated evidence, is what will be needed.

Secondly, that while we do believe that the consultative process followed in generating this list of priorities can assist in establishing a more coordinated strategy for research in the future, it is also clear that capacity for conducting such research will need to be grown. Close to 40% of our respondents, for example, indicated that they had never published in the field, although they may have done so in other disciplines. As mentioned in the introduction to this article, HPER outputs in the region are low. Indeed, one of the stated aims of the AFREhealth HPER TWG is to grow the community of active scholars in SSA and intentional steps towards such growth will be a necessary condition for implementing a research agenda. Nevertheless, we believe that this work provides a platform from which more focused, contextually relevant research questions can be developed and refined [29]. It could further be argued that strengthening research capacity in the region could also have value for health research generally – an area of critical need.

A final conundrum relates to the issue of funding and of convincing funders to invest in the region. Without funding it can be difficult to initiate the sort of multi-site, in-depth work that will lead to publications in leading journals. Without a proven track record and evidence of expertise, often measured in terms of publication outputs, funding applications are unlikely to be successful. Given that institutional funding for educational research in medicine is scarce and external grants are few and highly competitive, coordination of research efforts could potentially be of great value to the HPER community in SSA. In the New Zealand study mentioned earlier, Wilkinson and colleagues [28] expressed the hope that working collaboratively, sharing examples of best practice, and purposefully coordinating their research activities, would strengthen local research capacity while at the same time contributing to HPE scholarship globally. We trust that this work may similarly contribute to global debates, reminding us of the importance of context and relevance when embarking on research activities, on the one hand, and the responsibility of HPE researchers to generate evidence that can challenge or inform policies that may be constraining the training of health professionals, on the other.

### Strengths and limitations

We premised our choice of the Delphi approach on its relevance for HPE educators and ability to establish consensus on priorities [14]. The traditional idea of the Delphi technique, is to define, select, and engage a relatively homogenous group of experts throughout the process [13]. We modified this by engaging a larger and more diverse group for the two consensus building rounds. Involving respondents with divergent opinions increased the number of perspectives to be considered, and relative ‘newcomers’ could have contributed more novel opinions than those established in the discipline [30]. This may be the reason why our number one priority is of a wider contextual nature. On the other hand, this could also have limited the research priorities that emerged from the structured sub-fields of HPE.

Sustaining participation across rounds in a Delphi study is a known challenge [31]. Nonetheless, the outcomes of the process are strongly shaped by those most engaged, which is underlined by the fact that the number of individuals with more than five publications remained the same during both the consensus seeking round, meaning that those with more ‘expertise’ remained engaged through the two rounds. Another possible limitation is of course non-respondent bias. Consequently, there may be important research priorities for some settings that are not reflected here. The results of the study was based on the Delphi method only and could have been strengthened by some supporting qualitative data.

## Conclusion

To the best of our knowledge, this is the first study in SSA to explore HPER priorities for the region. We did so through leveraging the input of regional experts and researchers from diverse backgrounds, but with a shared interest in healthcare and the education thereof. The research was premised on the assumption that having a shared agenda could build evidence that is regionally relevant while facilitating the efficient and appropriate allocation of resources. Nested within this assumption is an acknowledgement that those of us who live and work in the region are best positioned to chart a way forward, and to resist hegemonic practices that are often externally imposed. Our challenge going forward will be to see the effective translation of this priority setting activity into education research policy and practice. Notwithstanding the challenges identified in terms of policy and funding, individual institutions in the region can reflect on these priorities as they seek to establish research strategies for themselves. Such strategies can intentionally look to engage regional partners as a first step to growing a larger network of HPE researchers across SSA.

## Supplementary Information

**Additional file 1.**

**Additional file 2.**

## Data Availability

The datasets and materials are available from the corresponding author on request.

## Abbreviations

AFREhealth: African Forum for Research and Education in Health
AfriPEN: The African Interprofessional Education Network
HPE: Health professions education
HPER: Health professions education research
ICT: Information communications technology
IPECP: Interprofessional education and collaborative practice
IRB: Institutional Review Board
MEPI: Medical Education Partnership Initiative
NEPI: Nursing Education Partnership Initiative
PEPFAR: President’s Emergency Plan for AIDS Relief
SSA: Sub-Saharan Africa
TWG: Technical Working Group
