# Supplementary material for: Identifying research priorities for health professions education research in sub-Saharan Africa using a modified Delphi method
Source: BMC Med Educ. 2020 Nov 18;20:443. doi: 10.1186/s12909-020-02367-z (PMC7672834; doi:10.1186/s12909-020-02367-z)
Supplement: Supplementary file 1 — Additional file 1. [file 12909_2020_2367_MOESM1_ESM.pdf]

Topic Generation Survey (Step/Survey One):

Consent at the beginning of the Qualtrics survey:

We are asking you to take part in a survey to help create and prioritize a health professions education research agenda for sub-Saharan Africa. This is specifically to do with research on how we educate and train the health workforce at pre-service and postgraduate levels. This survey is the first step where we elicit suggestions for potential topics. In subsequent rounds of the survey, participants will rank or prioritize suggested topics.

Participation in this survey is optional. Completion time will take about 5-10 minutes. You can skip questions that you do not want to answer or stop the survey at any time. We will keep your answers confidential and will not share your personal information with anyone outside the research team.

This study is certified as exempt by the University of California, San Francisco Institutional Review Board (#19-28050). The UCSF IRB can be reached with any questions at +001-415-476-1814.

Please contact Dr. Michael Reid at Xemail or Xphone if you have any questions about this survey.

Please click start to proceed with this survey.

What are potential research questions, topics, or areas of focus that should be a priority in a medical education research agenda for pre-service and post-graduate trainees in Sub-Saharan Africa (SSA)? Please fill in suggestions below.

Research Question or Agenda Item:
Please provide a short description of your understanding of this question/item:
Please provide a short description of what you understand under this research question / agenda item?
Why do you think it is important for SSA?

Research Question or Agenda Item (optional):
Please provide a short description of your understanding of this question/item (optional):
Why do you think it is important for SSA? (optional)

Research Question or Agenda Item (optional):
Please provide a short description of your understanding of this question/item (optional):
Why do you think it is important for SSA? (optional)

Please provide any other comments or suggestions.

ROUND ONE SURVEY

Survey to prioritize topic areas for health professions education research in SSA

AFREhealth and the University of California, San Francisco are working together to determine a research agenda for Health Professions Education (HPE) in sub-Saharan Africa (SSA). As a participant in the AFREhealth 2019 Symposium, we are asking you to take part in a survey to help create and prioritize such a health professions education research (HPER) agenda for SSA. The AFREhealth Technical Working Group in HPER has generated a list of research topics. In this survey you will be asked to rate each one based on how important you believe it would be to include in a HPER agenda for SSA.

Participation in this survey is optional. It will take about 10 minutes to complete. You can skip questions that you do not want to answer or stop the survey at any time. We will keep your answers confidential and will not share your personal information with anyone outside the research team. We will ask you to please share your email address with us as we intend to perform another round of the survey, in keeping with the Delphi technique, and your participation in that second round would be invaluable.

This study is certified as exempt by the University of California, San Francisco Institutional Review Board (#19-28050). The UCSF IRB can be reached with any questions at +001-415-476-1814. Please contact Dr. Michael Reid at Xemail or Xphone if you have any questions about this survey.

Demographics/Background

Please provide your email address __________________

Approximately how many years have you been working in your field? __________________

In which country do you primarily work? __________________

Are you a qualified health professional? (circle) YES NO

a. If yes, please mark in which health profession you are qualified. Please circle only one answer.

a. Dietician	d. Occupational therapist	g. Physiotherapist
b. Medical doctor	e. Pharmacist	h. Speech therapist
c. Nurse	f. Physician assistant	i. Other ______________

b. If no, please name your profession _________________

How would you describe your experience in HPER? Please circle only one answer.

a. Novice
b. Somewhat experienced
c. Experienced

How many HPE-related publications have you had? Please circle only one answer.

a. None
b. Less than 5

c. More than 5

<u>Content Prioritization</u>

Please rate how important you think each of the HPER topics listed in the table below is to include in a HPER agenda in SSA. Pick only one answer per HPER topic by selecting the box that best describes your rating of the objective.

Explanation of options:
Must be included: In my opinion this HPER topic is very important for SSA and must be included in the priority list
Could be included: In my opinion this HPER topic is somewhat important for SSA and could be included in the priority list, but it is not high priority in my opinion
Do not include: In my opinion this HPER topic is not important for SSA and should not be included in the priority list
No vote: I do not have enough knowledge of this topic to make an informed decision of its importance

	Research Topic	Must be included	Could be included	Do not include	No vote
1	Addressing the human resources for health challenges in rural and remote areas				
2	Social accountability strategies in response to the region's education transformation agenda				
3	Relevance of postgraduate education for SSA and beyond				
4	Interprofessional collaboration and practices in SSA				
5	Teaching a holistic and person-centered care approach				
6	Factors for effective distributed learning for health professional students				
7	Finding sufficient and suitable resources/settings for workplace-based/community-based training				
8	Assessment practices				
9	Integration of graduate competencies into undergraduate curricula				
10	Equipping students with self-regulation skills				
11	Models for understanding how change occurs in a health system				
12	Quality assurance processes and procedures in health professions education				
13	Tracking graduates into the health workforce				
14	Student retention				
15	Current status and challenges of health professions education research in SSA				
16	Relevance of communication skills training in culturally diverse contexts in SSA				
17	Potential of rural communities as platforms for training health care professionals				
18	Responsive curricula to the health needs of SSA				
19	Faculty development for clinical teaching				
20	Status of faculty development in SSA				

21	Using the ICF (International Classification of Functioning, Disability and Health) as an approach to person-centered student learning				
22	Capacity building efforts to strengthen HPE and the system in which it is conducted in SSA				
23	Ideal learning environments in SSA				
24	Trainee well-being, resilience, and stressors in health professions education				
25	Learner identity on how learners view their role in society in SSA				
26	Learner transition from high school to university				

Please add other topics you feel should be considered for research priorities that were not included above.

Please return this paper to the registration desk by the end of teatime on Friday morning, August 9, 2019.

ROUND TWO SURVEY

Survey to prioritize topic areas for health professions education research in SSA

AFREhealth is working to determine a research agenda for Health Professions Education (HPE) in sub-Saharan Africa (SSA). As you participated in the AFREhealth 2019 Symposium Delphi first round, we ask you again to help prioritize a health professions education research (HPER) agenda for SSA.

As a reminder, the AFREhealth Technical Working Group in HPER generated a list of research topics, and you ranked those. Thank you! In keeping with the Delphi technique and based on consensus criteria, the list of topics has been narrowed down. Since you kindly shared your email address, we ask you to again rate how important you believe each of these research topics is to include in a HPER agenda for SSA.

Participation in this survey is optional. It will take about 5 minutes to complete. You can skip questions that you do not want to answer or stop the survey at any time. We will keep your answers confidential and will not share your personal information with anyone outside the research team.

This study is certified as exempt by the University of California, San Francisco Institutional Review Board (#19-28050). The UCSF IRB can be reached with any questions at +001-415-476-1814. Please contact Dr. Michael Reid at Xemail or Xphone if you have any questions about this survey.

Demographics/Background

Please provide your email address __________________

Approximately how many years have you been working in your field? __________________

In which country do you primarily work? __________________

Are you a qualified health professional? (circle) YES NO

a. I**f yes, please mark in which health profession you are qualified. Please circle only one answer.**

a. Dietician	d. Occupational therapist	g. Physiotherapist
b. Medical doctor	e. Pharmacist	h. Speech therapist
c. Nurse	f. Physician assistant	i. Other ______________

b. **If no, please name your profession** _________________

How would you describe your experience in HPER? Please circle only one answer.

d. Novice
e. Somewhat experienced
f. Experienced

How many HPE-related publications have you had? Please circle only one answer.

d. None
e. Less than 5
f. More than 5

Content Prioritization

Please rate how important you think each of the HPER topics listed in the table below is to include in a HPER agenda in SSA. Pick only one answer per HPER topic by selecting the box that best describes your rating of the objective.

Explanation of options:
Must be included: In my opinion this HPER topic is very important for SSA and must be included in the priority list
Could be included: In my opinion this HPER topic is somewhat important for SSA and could be included in the priority list, but it is not high priority in my opinion
Do not include: In my opinion this HPER topic is not important for SSA and should not be included in the priority list
No vote: I do not have enough knowledge of this topic to make an informed decision of its importance

	Research Topic	Must be included	Could be included	Do not include	No vote
1	Addressing the human resources for health challenges in rural and remote areas				
2	Relevance of postgraduate education for SSA and beyond				
3	Interprofessional collaboration and practices in SSA				
4	Teaching a holistic and person-centered care approach				
5	Quality assurance processes and procedures in health professions education				
6	Current status and challenges of health professions education research in SSA				
7	Relevance of communication skills training in culturally diverse contexts in SSA				
8	Potential of rural communities as platforms for training health care professionals				
9	Responsive curricula to the health needs of SSA				
10	Faculty development for clinical teaching				
11	Theories that can strengthen HPE, including drivers of learner agency during workplace learning and alternative training models				
12	Resources, political commitment, and funding for HPE in SSA				
13	The role of information communications technology in HPE				
14	Training for mobility of health professionals across Africa				
