# Supplementary material for: Identifying research priorities for health professions education research in sub-Saharan Africa using a modified Delphi method
Source: BMC Med Educ. 2020 Nov 18;20:443. doi: 10.1186/s12909-020-02367-z (PMC7672834; doi:10.1186/s12909-020-02367-z)
Supplement: Supplementary file 2 — Additional file 2. [file 12909_2020_2367_MOESM2_ESM.docx]

**Supplementary Materials**

(1) See PDF of surveys (including topic generation, round one survey, and round two survey)

(2) Results from Round One

| Round One | | | | | |
| --- | --- | --- | --- | --- | --- |
| Topics | % rating topic ‘must include’ | % rating topic ‘could be included’ | % rating topic ‘do not include’ | ≥70% consensus met for ‘must include’? | Additional Topics suggested |
| Interprofessional collaboration and practices in SSA | 91 | 9 | 0 | Yes | Training for mobility of health professionals across Africa |
| Addressing the human resources for health challenges in rural and remote areas | 87 | 13 | 0 | Yes | Theories that can strengthen IPE, including drivers of learner agency during workplace learning and alternative training models |
| Relevance of communication skills training in culturally diverse contexts in SSA | 74 | 24 | 2 | Yes | The role of information communications technology in HPE |
| Responsive curricula to the health needs of SSA | 73 | 27 | 0 | Yes | Resources, political commitment, and funding for HPE in SSA |
| Relevance of postgraduate education for SSA and beyond | 72 | 25 | 3 | Yes |  |
| Quality assurance processes and procedures in health professions education | 72 | 26 | 2 | Yes |  |
| Current status and challenges of health professions education research in SSA | 72 | 27 | 1 | Yes |  |
| Faculty development for clinical teaching | 72 | 28 | 0 | Yes |  |
| Teaching a holistic and person-centered care approach | 71 | 25 | 4 | Yes |  |
| Potential of rural communities as platforms for training health care professionals | 70 | 29 | 1 | Yes |  |
| Trainee well-being, resilience, and stressors in health professions education | 69 | 29 | 2 | No |  |
| Integration of graduate competencies into undergraduate curricula | 68 | 27 | 5 | No |  |
| Assessment practices | 65 | 33 | 2 | No |  |
| Capacity building efforts to strengthen HPE and the system in which it is conducted in SSA | 64 | 31 | 5 | No |  |
| Social accountability strategies in response to the region’s education transformation agenda | 60 | 36 | 4 | No |  |
| Factors for effective distributed learning for health professional students | 60 | 37 | 3 | No |  |
| Finding sufficient and suitable resources/settings for workplace-based/community-based training | 60 | 37 | 3 | No |  |
| Ideal learning environments in SSA | 59 | 33 | 8 | No |  |
| Tracking graduates into the health workforce | 53 | 44 | 3 | No |  |
| Equipping students with self-regulation skills | 52 | 43 | 5 | No |  |
| Models for understanding how change occurs in a health system | 52 | 44 | 4 | No |  |
| Learner identity on how learners view their role in society in SSA | 47 | 47 | 6 | No |  |
| Learner transition from high school to university | 46 | 44 | 10 | No |  |
| Status of faculty development in SSA | 44 | 53 | 3 | No |  |
| Student retention | 43 | 50 | 7 | No |  |
| Using the ICF as an approach to person-centered student learning | 39 | 57 | 4 | No |  |

HPE = Health Professions Education; HPER = HPE Research; ICF

(3) Results from Round Two

| Round Two | | | | |
| --- | --- | --- | --- | --- |
| Topics | % rating topic ‘must include’ | % rating topic ‘could be included’ | % rating topic ‘do not include’ | ≥70% consensus met for ‘must include’? |
| Addressing the human resources for health challenges in rural and remote areas | 98 | 2 | 0 | Yes |
| Interprofessional collaboration and practices in SSA | 95 | 4 | 1 | Yes |
| Teaching a holistic and person-centered care approach | 92 | 8 | 0 | Yes |
| The role of information communications technology in HPE | 85 | 11 | 4 | Yes |
| Faculty development for clinical teaching | 82 | 18 | 0 | Yes |
| Quality assurance processes and procedures in health professions education | 80 | 20 | 0 | Yes |
| Resources, political commitment, and funding for HPE in SSA | 76 | 24 | 0 | Yes |
| Responsive curricula to the health needs of SSA | 75 | 25 | 0 | Yes |
| Potential of rural communities as platforms for training health care professionals | 71 | 27 | 2 | Yes |
| Relevance of communication skills | 70 | 28 | 2 | Yes |
| Relevance of postgraduate education for SSA and beyond | 69 | 29 | 2 | No |
| Current status and challenges of HPER in SSA | 69 | 25 | 6 | No |
| Theories that can strengthen HPE, including drivers of learner agency during workplace learning and alternative training models | 58 | 40 | 2 | No |
| Training for mobility of health professionals across Africa | 54 | 39 | 7 | No |
